# Do preschools differ in promoting children’s physical activity? An instrument for the assessment of preschool physical activity programmes

**DOI:** 10.1186/1471-2458-13-795

**Published:** 2013-09-03

**Authors:** Elena Sterdt, Natalie Pape, Silke Kramer, Michael Urban, Rolf Werning, Ulla Walter

**Affiliations:** 1 Institute for Epidemiology, Social Medicine and Health Systems Research, Hannover Medical School, Carl-Neuberg-Str.1, D-30625 Hanover, Germany; 2 Institute for Special Education, Leibniz University Hannover, Hanover, Germany; 3 Faculty of Educational Science, Bielefeld University, Bielefeld, Germany

**Keywords:** Physical activity, Preschool, Children, Assessment instrument

## Abstract

**Background:**

Preschools offer high potential for preventive interventions. However, little is known about the structure of preschool programmes to promote physical activity (PA) in preschoolers although almost all children aged three to six years spend one third of the day at preschool. The aim of this study was to determine whether and to what extent preschools implement systematic PA promotion measures using an instrument specifically developed to assess and systematize preschool PA programmes.

**Methods:**

In the cross-sectional study a baseline survey of preschool education policies was conducted to identify and assess the type and extent of PA programmes and opportunities in preschools in the State of Lower Saxony, Germany. An assessment instrument was developed to identify preschools with systematic PA programmes (type 1) and those without PA programmes (type 2) based on the following quality criteria: A) written PA policy, B) structured weekly PA offerings for all children; C) at least one qualified physical education teacher; D) PA-friendly indoor and outdoor facilities (exercise room, situational PA opportunities, outdoor areas, play equipment etc.), and E) structured PA promotion in place for at least two years. A third type of preschool that promotes PA in children to some extent (i.e., that meets the criteria partially but not completely) was classified as “preschools with limited PA programmes”.

**Results:**

2415 preschools participated in the survey (response rate: 59%). The results show that 26% (n = 554) have a systematic PA programme while 3% (n = 64) have no PA programme. Most (71%, n = 1514) were classified as limited PA programme preschools. All three types of preschools differed significantly (p = .000) from each other in terms of size (small vs. large). Most of the preschools without PA programmes are small half-day preschools.

**Conclusions:**

The study investigated an assessment-instrument providing extensive insight into the nature, extent and routine practical implementation of PA promotion in preschools. The criteria used to evaluate preschool PA programmes are well-suited to identify the different preschool PA programme types and target areas in the field of PA promotion in which specific measures (teacher education, structured PA offerings, etc.) can be implemented in future interventions.

## Background

Sports and physical activity (PA) in childhood promotes mental health and well-being [1,2]. Preschool age (three to six years) is a critical period in the development of a healthy lifestyle including, in particular, PA behaviour. At this early age, children should engage in PA through structured and unstructured play [3]. As the first stage of the education system, preschool is an ideal place to promote PA [4] and offers high potential for preventive PA interventions. Therefore, PA should be a central part of the preschool educational curriculum [5].

Almost all children between the ages of three and six years spend one-third of their day at preschool. Although this applies to 93% of all three- to six-year-olds in Germany [6], few studies to date have investigated the impact of preschool on PA in children [7]. It is assumed that structured and unstructured PA opportunities, PA-friendly environments, and teachers with PE training promote PA in preschool children [8-10].

Each of the 16 German states issues its own guidelines governing the content of preschool programmes and curricula. In all 16 states, health forms a separate area of the curriculum, usually in the context of exercise and movement. Nine of the 16 curricula include “body, physical exercise and health” as a combined topic area. There are, however, significant differences in the scope and integration of health education areas [11].

One study showed that although PA is one of the most frequent activities (97%) at nearly all preschools studies (n = 643), it represents a fundamental working concept at only 27% [12]. One cannot infer from the published details whether these were systematic PA measures implemented on a regular basis and on a defined time scale (i.e., a comprehensive package of PA measures), or whether they were singular PA measures.

Although preschool offers a variety of behavioural and environmental opportunities for the promotion of PA in children, little is known about the structure of such opportunities at preschools [4,12,13]. Therefore, it would be useful to have systematic studies of preschool physical activity programmes (PAPs) and opportunities and of factors that influence the PA of children [14]. The present study is the first to provide a comprehensive assessment of preschools and preschool education programmes in one German state.

In Germany, there are no uniform criteria for assessing the quality of prevention and health promotion in preschool education [12]. However, three German states (Lower Saxony, North Rhine-Westphalia and Rhineland-Palatinate) have already developed evidence-based guidelines for preschool certification in the field of PA [5]. All three guidelines include different quality areas to assess the extent to which systematic PA promotion is integrated in preschool everyday life. Central quality areas include: A) PA policy, B) individual structured PA offerings, C) teacher qualification and D) structural conditions/facilities.

In this research project, an instrument for the comprehensive consideration and analysis of the four quality areas was developed to assess and systematize preschool PAPs. The aim of the study is to analyse the type and extent of PAPs being implemented in preschools in Lower Saxony, Germany.

## Methods

In the cross-sectional study a baseline survey of preschool education policies was conducted to identify and assess the type and extent of preschool PAPs and opportunities. Prior ethical approval was obtained from the Ethics Committee of the Hannover Medical School (Approval No. 6004).

The baseline survey (February to April 2011) consisted of a comprehensive online and postal survey of programmes and opportunities at all preschools (N = 4114) in Lower Saxony, the second largest and the fourth most populous state in Germany. Data on structural factors (size, ownership, day-care hours, etc.), socio-demographic factors (e.g. location, migration background and socioeconomic status (SES) of the children attending preschool), education policy, health education policy, PA measures and opportunities, social interactions, prevention and health promotion, and quality assurance were collected by questionnaire.

The five-page preschool survey was to be conducted mainly online. As it was known from our previous studies that some of the preschools had no access to the Internet and/or had technical difficulties answering online questionnaires, preschools that did not respond to the survey online were sent the questionnaire by regular mail.

Our goal was to make the survey as quick and easy to complete as possible in order to achieve the highest possible response rate. Pre-testing at 20 preschools showed that it takes about five to ten minutes to complete the questionnaire. The instrument was designed to allow the child care centre directors to answer the questions with assistance from key staff members.

The baseline survey provided a base of data for the identification of preschools with systematic PAPs (type 1) and no PAPs (type 2). Systematic PAPs were defined as integrated, comprehensive and targeted PA promotion programmes. An assessment instrument was developed to identify preschools with systematic PAPs based on the following five quality criteria, comprising a total of 22 items:

A) Written PA policy (two items): written educational concept and/or programme including the topic of PA promotion and describing the preschool’s policies and practices regarding PA;

B) Structured weekly PA offerings for all children (three items): at least 120 minutes of structured physical activities per week, with at least 75% of children participating;

C) At least one trained physical education (PE) teacher (two items): at least one teacher with additional qualifications in PE (e.g. coach) who regularly participates in continuing education courses (at least once every two years);

D) PA-friendly indoor and outdoor facilities (14 items): exercise room, situational PA opportunities, freely designed outdoor areas, play equipment, etc.;

E) Structured PA promotion in place for at least two years (one item).

The selection criteria were based on evidence-based German guidelines for preschool certification in the field of PA in three German states (Lower Saxony, North Rhine-Westphalia and Rhineland-Palatinate) [5]. As all three guidelines specify four quality areas (PA policy, individual structured PA offerings, teacher qualification, and structural conditions/facilities), these areas were included in our assessment instrument. They also had to be amenable to questionnaire measurement.

All items of each criterion had to be met to get one point for each criterion, corresponding to a maximum score of five. Preschools with systematic PAPs had to score at least four out of five points. All item requirements for Criteria A, C and E and ten of 14 for Criterion D had to be met. Regarding Criterion B, it must be noted that some preschools were also classified as having systematic PAPs if they implemented structured physical activities for only 90 to under 120 minutes per week. In this case, 0.3 points were deducted from the score for this criterion.

Criterion E involved the question: For how many years has structured PA promotion been implemented at your facility? In order to receive one point for this item, structured PA promotion had to have been implemented at the facility for an extended period (at least two years).

By definition, preschools with no PAPs (type 2) had a) no structured PA offerings and b) no trained PE teachers, so they could only receive a maximum of two points for the criteria A) written PA policy and D) PA-friendly indoor and outdoor facilities. Preschools reaching a score of 2 to 4 points were classified as preschools with limited PAPs. These preschools promote PA in children to some extent, i.e., they meet the criteria partially but not completely.

In addition to the items of the assessment instrument, we surveyed the frequency of PA promotion measures in ongoing educational work. In the end, this item was not included in the assessment instrument because specific types of structured or unstructured activities were not specified.

The preschools were asked to report the rate of specific promotion of social interaction in daily preschool routine. Social interaction was defined as “action and communication among preschool children”. Two examples of specific measures to promote social interaction were “conflict management/prevention of violence” and “promotion of socio-emotional development”.

The presence of cooperation with a professional provider of PA promotion measures (e.g. sport club) was also determined.

The preschools were also asked to provide information on the socio-demographic characteristics of their area, that is, to indicate whether they are located in a deprived area and how many of their children have a low socioeconomic status (SES) and/or migration background. The proportion of children with a low SES was determined based on the question: How many children at your facility are exempt from paying preschool fees? In Germany, parents exempt from paying preschool fees, receive social welfare benefits. Children with a migration background were defined as those in which both parents have a migration background.

Respondents were also asked to identify their function at the preschool (director, assistant director, teacher or member of the board).

Data analysis was performed using SPSS 20.0 for Windows. The quantitative data analysis was performed using primarily descriptive statistics. Differences in preschool characteristics (size, day care hours, socio-demographic background, etc.) were tested using chi-square statistics. An alpha level of 0.05 was used to judge statistical significance.

## Results

2415 preschools participated in the baseline survey, corresponding to a return rate of 59%. Of the participating preschools, 34% (n = 826) responded online, and 66% (n = 1593) responded by regular mail. The return rate was doubled by combining the online and postal surveys. In the vast majority of cases (87%), the questionnaires were completed by the preschool director. The rest were submitted by the assistant director (6%), a teacher (5%), or a member of the board (1%). 2132 questionnaires were included in the analysis and 287 were excluded due to incomplete or missing responses to items of the assessment instrument.

The results show that 26% (n = 554) of the preschools surveyed have systematic PAPs, and 3% (n = 64) have no PAPs. Most (71%, n = 1514) promote PA in children to some extent, and were thus classified as limited PAP preschools.

### Fulfilment of quality criteria

*Preschools with systematic PAPs* achieved an average score of 4.5. Only 2.5% (n = 14) met all criteria of the assessment instrument and achieved the maximum score of five points. To be classified as a preschool with a systematic PAP, the preschool had to meet the following three requirements: written PA policy, at least one trained PE teacher, and structured PA promotion in place for at least two years. 12% (n = 64) of preschools with systematic PAPs completely fulfilled those for B) Structured weekly PA offerings for all children, and 15% (n = 85) completely fulfilled the requirements for D) PA-friendly indoor and outdoor facilities. The majority of preschools (88%) implemented structured PA offerings for 90 to 120 minutes per week.

*Preschools with limited PAPs* achieved an average score of 3.5. Analysis to identify the largest group of preschool PAP types in terms of the degree of fulfilment of a single criterion of the assessment instrument showed that almost all preschools (96%, n = 1351) have a written policy for the promotion of PA. 58% (n = 785) of the preschools have been implementing structured PA offerings for at least 90 minutes per week for more than two years. 72% (n = 1078) of the preschools of this type fulfilled at least ten of 14 items for the criterion PA-friendly indoor and outdoor facilities. Just under one-third of the preschools had at least one teacher with additional qualifications in the field of PE who regularly attended continuing education courses.

*Preschools without PAPs* achieved an average score of 1.4. The majority of preschools (84%, n = 47) received one point for the criterion written PA policy. 42% (n = 26) of the preschools also met at least ten of the 14 item requirements for the criterion PA-friendly indoor and outdoor facilities (Table 1).

**Table 1:** Description of preschool types by fulfillment of quality criteria, structural conditions and socio-demographic characteristics

**Preschool type description**		**Preschools with systematic**	**Preschools with limited**	**Preschools without**
		**PAPs (n = 554)**	**PAPs (n = 1514)**	**PAPs (n = 64)**
**Fulfillment of quality criteria**				
Average score (out of five)		4.5	3.5	1.4
A: Written PA policy		100%	95.5%	83.9%
B: Structured weekly PA offerings	90 – 120 min	88.4%	55.2%	0%
	> 120 min	11.6%	2.5%	0%
C: At least one trained PE teacher		100%	32.4%	0%
D: PA-friendly facilities	10 of 14 items	84.7%	60.9%	40.3%
	14 of 14 items	15.3%	10.6%	1.6%
E: Structured PA promotion for at least 2 years		100%	86.0%	0%
**Structural conditions**				
Size (number of students and teachers)	< 40 children	18.9%	28.8%	57.8%
	> 80 children	45.7%	35.5%	14.1%
	< 5 teacher	19.0%	33.0%	67.2%
	> 10 teacher	43.4%	27.5%	12.5%
Ownership	Municipality	31.3%	36.1%	29.7%
	Church	36.9%	34.4%	31.3%
	Non-statutory welfare	16.5%	13.8%	3.1%
	Parent association/initiative	11.0%	13.4%	35.9%
Day care hours*	Full-day care	56.4%	48.3%	32.8%
	Two-thirds care	49.0%	45.4%	32.8%
	Half-day care	78.7%	80.0%	84.4%
**Socio-demographic characteristics**				
Children with low SES	< 25%	61.5%	65.5%	69.5%
	> 75%	5.9%	4.0%	3.4%
Children with migration background	< 25%	75.9%	77.0%	74.6
	> 75%	2.1%	2.1%	1.7%
Deprived area		10.8%	11.6%	12.5%

*PAP* physical activity programme*, SES* socioeconomic status; *The percentages shown include multiple answers because the facilities often offer several different forms of child care depending on the needs of the parents.

### Structural conditions

Analysis of the structural conditions of the preschools showed that the majority of preschools with no PAPs are small sites with less than 40 children (58%; n = 37), compared to 19% (n = 105) and 29% (n = 434) of those with systematic and limited PAPs, respectively. Analysis by the number of teachers employed at the facilities shows that the majority of preschools without PAPs (67%, n = 43) employed fewer than five teachers, whereas just under one-fifth (19%, n = 105) of preschools with PAPs employed less than five teachers (Table 1). All three types of preschools differed significantly (p = .000) in terms of size (small vs. large) from each other.

Analysis by ownership revealed that the preschools are most commonly owned by municipalities, churches, non-statutory welfare organizations, and parent associations/initiatives. The proportion of municipal and church ownership of preschools was evenly distributed (approximately one-third in all three groups). Preschools without PAPs are most commonly owned by parent associations/initiatives 36%, n = 23), whereas the other two types of preschools are least commonly owned by such groups (11%, n = 61 and 13%, n = 202, respectively). Preschools with systematic PAPs were most commonly owned by churches (37%, n = 204), and preschools with limited PAPs by municipalities (36%, n = 545) (Table 1).

Analysis according to the number of day care hours showed that full-day child care is provided by 56% (n = 312) of the preschools with systematic PAPs, 48% (n = 730) of those with limited PAPs and one-third (n = 21) of those with no PAPs (Table 1). Frequently, different forms of day care are offered depending on the needs of parents. The majority of preschools offer half-day care (four hours). Two-thirds day care is equivalent to six hours of care per day, and full day care is equivalent to eight hours/day. There were significant differences in terms of number of day care hours between preschools without PAPs and preschools with systematic PAPs (p = .000) and limited PAPs (p = .015).

### Socio-demographic characteristics

The three preschool types did not significantly differ from each other in terms of the socio-demographic background of the children attending preschool (SES and migration background) (p > .05). At the majority of preschools of all three PAP types (about 75% in each case), the percentage of children with a migration background was less than 25%. This also applied to the proportion of children of low SES, which was below 25% in approximately two-thirds of the preschools of each PAP type. Analysis according to location in a socially disadvantaged area showed no significant differences between the three preschool types (p > .05) (Table 1).

### PA promotion activities

**Figure 1:**
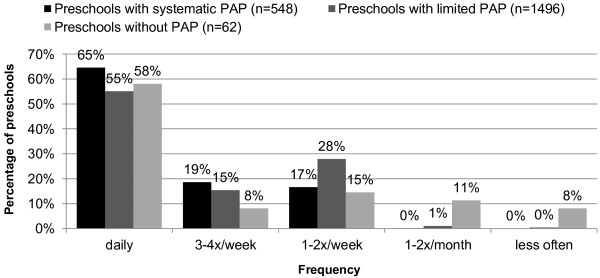
**Frequency of PA promotion measures in ongoing educational work.***PAP*, physical activity programme.

Analysis according to the frequency of PA promotion measures in current educational work showed that such measures are most frequently implemented on a daily basis at preschools with systematic PAPs (65%). However, more than half (58%) of the preschools without PAPs reported that they implement PA promotion measures on a daily basis. Compared to the other two types, preschools lacking PAPs were much more likely to implement PA promotion measures infrequently, i.e., only once or twice a month (11%) or even less frequently (8%) (Figure 1).

**Figure 2:**
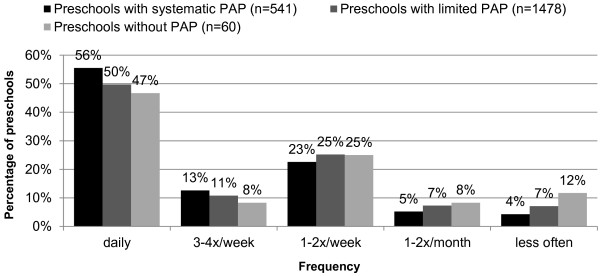
**Frequency of specific measures to promote social interaction in ongoing educational work (e.g. conflict management/prevention of violence, socio-emotional promotion).***PAP,* physical activity programme.

Analysis according to the frequency of specific measures to promote social interaction revealed no differences between the three preschool types. Such measures were most frequently implemented at preschools with systematic PAP (56%) compared to slightly less than half (47%) of preschools with no PAP (Figure 2).

**Figure 3:**
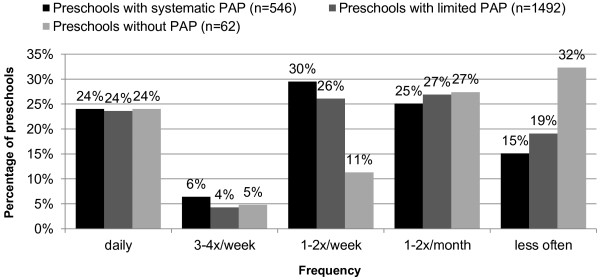
**Frequency of the use of an outdoor area outside the preschool premises.***PAP,* physical activity programme.

24% of preschools of all three types use an outdoor area (park, forest, playground, etc.) outside the preschool premises on a daily basis. Preschools with systematic and limited PAPs had similar rates of outdoor area use. One-third of the preschools without a PAP rarely made use of outdoor areas (Figure 3).

Preschools with systematic and limited PAPs showed similar rates of use of external facilities (swimming pools, gyms, etc.), whereas those lacking a PAP used external facilities much less frequently. In fact, nearly half of the non-PAP preschools (47%) reported that they never visit external facilities.

Regarding networking activity, one-third of all preschools with systematic PAPs reported that they cooperate with a sports club compared to 22% of those with limited PAPs and 8% of those without PAPs.

## Discussion

The present article reports on the results of our assessment of the type and extent of preschool PAPs in Lower Saxony, Germany. The assessment instrument developed in the scope of this study can be used to check whether and to what extent preschools implement systematic PA promotion, i.e., integrated comprehensive and targeted PAPs. The present study was the first study of this design conducted in Germany.

In Germany, there are no uniform strategies, principles, programmes or quality criteria for designing effective and efficient health promotion, not even in the field of PA [12]. The assessment tool assessed preschools’ PAPs based on different German state guidelines for preschool certification in the field of PA [5]. Therefore, the instrument can be used for nationwide preschool PAP assessment.

The assessment instrument examines four key quality areas: A) PA policy, B) individual structured PA offerings, C) teacher qualifications and D) structural conditions/facilities. In particular, the two quality areas B) individual structured PA offerings and C) teacher qualifications provide information about the extent to which a systematic PAP is integrated into the daily preschool routine. Therefore, the absence of structured PA offerings and the lack of trained PE teachers was the basis for defining the term “preschools without PAPs”.

Criterion E) “structured PA promotion in place for at least two years” was additionally modified to allow for the additional time it takes to implement and entrench structured PA promotion in daily preschool routine. This ensured that all parties involved (employees, children, parents, etc.) have sufficient time to familiarize themselves with the concept. In addition, there is evidence of the sustained implementation of a systematic PAP.

The Nutrition and Physical Activity Self-assessment for Child Care (NAP SACC) intervention was a similar study conducted in North Carolina, USA [15,16]. In the NAP SACC, best practice guidelines for a self-assessment instrument were developed based on the best available evidence to assess PA and nutrition policies and practices in child care settings. The investigators identified key PA areas (PA opportunities, play equipment, PA training/education for children and teacher, PA policies, etc.), which were similar to the criteria of the assessment instrument used in the present study.

The assessment criteria used in this study are well-suited to identify the different preschool PAP types and specific target areas in the field of PA promotion in which specific measures (teacher education, structured PA offerings, etc.) can be implemented in future interventions. Additional criteria are needed for greater differentiation between the different preschool PAP types. Moreover, further criteria are required for differentiation, in particular, of preschools with limited PAPs – the most common type.

Our instrument has some limitations. The responses of preschool directors regarding PAP in their preschools may be positively biased. However, this is the first large-scale assessment of preschool PAPs in Lower Saxony with a high response rate (59%). Our instrument provides a low cost survey method to evaluate PAPs in preschools with minimal respondent burden (5–10 minutes).

Because of the high return rate achieved in the full baseline survey, the results can be generalized to the total population of preschools in Lower Saxony, Germany. The high response rate to the postal questionnaire confirms our assumption that postal surveys are still the survey format preferred by preschools. The present study included preschools with a range of different facility characteristics, such as urban or rural location, SES, type of ownership, facility size, and working practices and policies. Therefore, all key subgroups are represented in the sample. The extent to which the findings are transferrable to other states can only be speculated because the framework conditions and education programmes for child care vary between states.

The results of the baseline survey showed that only a small percentage (3%, n = 64) of the investigated preschools have no PAP. However, only about one-quarter (26%, n = 556) of the preschools had systematic PAPs and 71% (n = 1514) had limited PAPs. Selection bias might be the reason why only a small number of preschools without a PAP participated. It is conceivable that preschools with comprehensive PA promotion activities might have been the predominant type participating in the baseline survey. However, a nationwide study also found that PA promotion was a basic working principle and practice at only about one-quarter of all preschools surveyed [12].

Overall, it is positive that the majority of preschools surveyed are already implementing PA promotion in children, but the scope and preschools varied greatly in the degree of systematic implementation of these activities. The reasons for the differences in implementation of PAPs in preschools are varied and can only be speculated based on the results.

### Socio-demographic characteristics

First, one can assume that socio-demographic characteristics, such as preschool location and migration background or the SES of preschool children, did not play a central role in the implementation of systematic PAPs because there were no significant differences between the three types of preschools in this respect. As a caveat, it must be noted that there were very large differences in group size between the three preschool types. Therefore, the results of the chi-square test must be interpreted cautiously.

### Quality areas

#### PA policy

The majority of preschools reported having a written educational concept including the topic of PA promotion; this also applies to the preschools without PAPs. This suggests that the presence of a written PA concept does not necessarily mean that a preschool will implement a systematic PAP in daily practice. Therefore, this measure cannot be used as a stand-alone criterion for evaluation of the implementation of a systematic PAP in preschool daily routine. The implementation of a quality management system is recommended to ensure that preschools can regularly evaluate their progress towards achieving the goals they set for themselves [12].

#### Individual structured PA offerings

When developing the assessment instrument, we also focused on the structured PA measures offered by the preschools. A systematic review by Ward et al. [9] showed that structured PA results in improved motor skills and increased activity levels in children.

However, increased activity levels were only achieved by significantly increasing the time for structured activity to at least 2.5 hours per week. The National Association for Sport and Physical Education (NASPE) recommends at least 60 minutes of structured activity per day for preschoolers [3], but these recommendations do not specifically apply to the childcare setting.

It is doubtful whether preschools can afford to provide such a large amount of structured PA considering the present finding that only a few preschools with systematic PAPs offered more than 120 minutes of structured PA per week. Still, structured PA at preschool can help to ensure that children reach the recommended levels of PA [4].

Although lacking structured PA, about half of all preschools without PAPs reported implementing daily measures to promote PA in their ongoing educational work. These measures probably consist of individual, unstructured activities. Further research is needed to identify the exact type of activity in question and to determine whether and to what extent these activities can be implemented on a regular and structured basis.

However, it is important to remember that structured PA should not be implemented at the expense of time for free play, which promotes the joy of PA, creativity and social interaction [17,18].

It is positive to note that in all three PAP groups, the majority of preschools selectively promoted the social interaction of children at least once or twice per week in a targeted manner. Preschools without PAPs conducted such measures least frequently, which also reflects the lack of an appropriate programme. Because PA in early childhood can mediate basic cognitive, emotional and social learning processes [7], social skills can be promoted in a targeted manner in the scope of PA promotion measures. This also applies to other educational goals, such as the promotion of cognitive and emotional skills.

#### Teacher qualifications

The behaviour, attitudes and knowledge of teachers are other key factors that influence the quality and quantity of PA promotion in preschools. Systematic reviews [7-9,19] showed that the activity levels of children are higher when their teachers demonstrate active PA behaviour and have relevant qualifications (higher levels of education, additional training, etc.). As teachers are key figures in preschool settings, they in particular must develop the knowledge and skills needed to support healthy education processes through PA promotion [5,20].

When implementing a fixed PAP in a heterogeneous group of preschool children, the teacher must be aware of the needs of each individual child. To achieve this, special and repeated teacher training is essential [8].

The results of the present study showed that only one-third of all preschools with limited PAPs (the largest group) have at least one teacher with additional qualifications in PE who regularly attends continuing education courses. A possible reason for this is that a lack of human resources, especially at smaller preschools, might hinder the teachers from participating in qualification courses. Policies and future interventions must place special focus on teacher qualification measures, such as training and continuing education courses [6].

Cooperation with professional PE providers, such as sports clubs, can help to increase the quantity and quality of PA promotion in preschools, even if no qualified teacher is available. However, only one-third of all preschools with systematic PAPs and just under one-fourth of those with limited PAPs reported that they cooperate with a sports club. Future interventions should ensure the stronger promotion of preschool support networks, particularly as this would also enhance the sustainability of PA promotion interventions [20].

#### Structural conditions/facilities

Compared to the other two types, the majority of preschools without PAPs were fairly small low-capacity facilities with low day care capacities and hours. Moreover, less than half of the preschools without PAPs had PA-friendly facilities. Other studies suggest that site-specific conditions (size, facilities/equipment, financial and human resources) influence the implementation of systematic PAPs [7-10].

One study that analysed the physical environment showed that generously designed interior and exterior preschool spaces have a positive effect on the level of PA in children [21]. Likewise, the presence of play equipment results in higher PA levels, improved physical fitness, and less sedentary activity in children. A particularly interesting finding was that portable play equipment (e.g. balls) has a positive effect on PA, whereas fixed play equipment seems to have no significant impact [7-9]. This is positive because portable play equipment is much cheaper to procure than permanently installed equipment such as climbing frames [8].

Some preschools, especially small ones, are limited in their capacity to install fixed equipment. It can be assumed that facilities with no PAP have few resources to spend on spaces and equipment, which is a major hurdle in the implementation of systematic PA promotion.

Facility-related factors such as size and amenities are difficult to change during the course of a short-term intervention. Nearby outdoor areas (parks, forests, playgrounds, etc.) or external facilities (swimming pools, gyms, etc.) could be utilized by preschools with limited indoor and outdoor capacities for PA promotion purposes. However, the preschools without PAPs tended to use outdoor areas and external facilities much less often than the other two types of preschools.

Future interventions should therefore be performed to determine which environmental conditions are present at preschools without PAPs and how they can be used effectively. The reasons why preschools without PAPs rarely use existing facilities in the vicinity must also be determined.

Nevertheless, it must be remembered that the responsibility for providing adequate resources for PA-friendly facilities lies mainly in the hands of the preschool owners. Preschools without PAPs are most commonly owned by parent associations/initiatives. It can be assumed that such owner groups have fewer financial and human resources than others, such as municipalities and churches. Still, one-third of the preschools without PAPs were owned by municipalities and churches, respectively. Therefore, the owners should also be made aware of the importance of PA in childhood.

The extent to which these key PA areas influence the PA behaviour of children either individually or collectively has not yet been sufficiently explored [9,15]. Further studies are needed to determine whether the criteria established in the present study are appropriate for differentiated assessment of preschool PAP. It would be advisable to include more detailed criteria such as the type of teacher qualifications in the field of PE and the implementation of structured PA opportunities in daily preschool routine.

However, the assessment-instrument used in the present study provides extensive insight into the nature, extent and routine practical implementation of PA promotion in preschools. Based on the results of the present study, it can be assumed that interventions for PA promotion in preschools should place special emphasis on preschools without PAPs. Ways in which these preschools can more strongly integrate systematic PAPs in daily preschool routine in spite of the potential lack of resources must be determined. When analysing preschools with limited PAPs, researchers can use the assessment instrument to identify target areas with deficits and initiate appropriate measures (teacher training, increasing the amount of structured activity, etc.). This may be helpful for future interventions.

The focus of interventions should not be solely on a defined quality area, such as individual structured PA offerings (as is often the case). Instead, all the key quality areas should receive equal consideration in order to achieve sustainable and lasting implementation of systematic PAPs in preschools.

## Conclusions

Further research is needed to determine which specific health-related design criteria are suitable for preventive activities in this field and to identify conditions that have a positive influence on PA in children in the preschool setting [12].

Planning, implementation and guidance of PA promotion in preschools and kindergartens is difficult due to the diversity of ownership, training types, curricula and participating institutions. In-depth consideration of PA and health as subjects in the state curricula is a step in the right direction [20]. The existing curricula should provide more concrete advice on the practical implementation of systematic PA promotion in preschools while taking differences in framework conditions into account.

The responsibility for the implementation of systematic PA promotion should not lie solely with the preschool directors and teachers, but also with the preschool owners and at national policy makers. Appropriate framework conditions must be established and sufficient financial, material and human resources made available to the preschools. Preschool is a central institution in early child care and education. As such, its potentials to promote the health and health status of children should be tapped more strongly in the future.

## Consent

Written informed consent was obtained from the teachers/parents/next of kin for the publication of this report and any accompanying images.

## Abbreviations

PA: Physical activity; PAP: Physical activity programme; PE: Physical education; SES: Socioeconomic status.

## Competing interests

The authors declare that they have no competing interests.

## Authors’ contributions

ES and NP contributed to the design of the study and its coordination and drafted the manuscript. ES and SK performed the baseline survey of preschools, the survey of children and parents, and the statistical analysis. NP carried out the ethnographic case studies and evaluated the ethnographic observations. MU, RW and UW contributed to the conception and design of the study, developed the assessment instruments, critically reviewed the manuscript, and approved the final version of the manuscript. All authors read and approved the final version of the manuscript.

## Pre-publication history

The pre-publication history for this paper can be accessed here:

http://www.biomedcentral.com/1471-2458/13/795/prepub

## References

[B1] Timmons B Naylor P Pfeiffer K Physical activity for preschool children - how much and how? Appl Physiol Nutr Metab 2007 32 S122 S134 18213943

[B2] McWilliams C Ball SC Benjamin SE Hales D Vaughn A Ward DS Best-practice guidelines for physical activity at child care Pediatrics 2009 124 1650 1659 10.1542/peds.2009-0952 19917582

[B3] NASPE National Association for Sport and Physical Education Active Start: A Statement of Physical Activity Guidelines for Children from Birth to Age 5 2009 2 Sewickley, PA: American Alliance for Health, Physical Education, Recreation, and Dance

[B4] Ward DS Physical activity in young children: the role of child care Med Sci Sports Exerc 2010 42 499 501 20068500 10.1249/MSS.0b013e3181ce9f85

[B5] Detert D Selchow U Balster K Beckmann U Heller J Hensler N Zertifizierter Bewegungskindergarten – Umsetzung in den Ländern Niedersachsen, Nordrhein-Westfalen und Rheinland-Pfalz [Certified physical activity kindergarten – implementation in the states Lower Saxony, North Rhine-Westphalia and Rhineland-Palatinate] Haltung und Bewegung 2007 27 19 27

[B6] Statistisches Bundesamt Statistiken der Kinder- und Jugendhilfe. Kinder und tätige Personen in Tageseinrichtungen und in öffentlich geförderter Kindertagespflege am 01.03.2012 Statistics of child and youth services. Children and staff in day care at the 01/03/2012 2012 Wiesbaden: Statistisches Bundesamt [https://www.destatis.de/DE/Publikationen/Thematisch/Soziales/KinderJugendhilfe/TageseinrichtungenKindertagespflege5225402127004.pdf?__blob=publicationFile]

[B7] Trost SG Ward DS Senso M Effects of child care policy and environment on physical activity Med Sci Sports Exerc 2010 42 520 525 20068496 10.1249/MSS.0b013e3181cea3ef

[B8] Kreichauf S Wildgruber A Krombholz H Gibson EL Vogele C Nixon CA Douthwaite W Moore HJ Manios Y Summerbell CD Critical narrative review to identify educational strategies promoting physical activity in preschool Obes Rev 2012 13 Suppl 1 96 105 22309068 10.1111/j.1467-789X.2011.00973.x

[B9] Ward DS Vaughn A McWilliams C Hales D Interventions for increasing physical activity at child care Med Sci Sports Exerc 2010 42 526 534 20068495 10.1249/MSS.0b013e3181cea406

[B10] Swinburn BA Sacks G Hall KD McPherson K Finegood DT Moodie ML Gortmaker SL The global obesity pandemic: shaped by global drivers and local environments Lancet 2011 378 804 814 10.1016/S0140-6736(11)60813-1 21872749

[B11] Friedrich T Die Bedeutung von Prävention und Gesundheitsförderung in Kindertageseinrichtungen. Expertise zum 13. Kinder-und Jugendbericht The relevance of prevention and health promotion in preschools. Expertise for the 13^th^ child and youth report 2009 Edited by Sachverständigenkommission des 13. Kinder- und Jugendberichts [http://ww.dji.de/bibs/13_KJB_Expertise_Friederich_Kita.pdf]

[B12] Kliche T Gesell S Nyenhuis N Bodansky A Deu A Linde K Neuhaus M Post M Weitkamp K Töppich J Koch U Prävention und Gesundheitsförderung in Kindertagesstätten. Eine Studie zu Determinanten, Verbreitung und Methoden für Kinder und Mitarbeiterinnen [Prevention and health promotion in preschools. A study on determinants, dissemination and methods for children and staff] 2008 Juventa Verlag: Weinheim und München

[B13] Finch M Wolfenden L Morgan PJ Freund M Wyse R Wiggers J A cluster randomised trial to evaluate a physical activity intervention among 3–5 year old children attending long day care services: study protocol BMC Publ Health 2010 10 534 10.1186/1471-2458-10-534 PMC2944368 20822543

[B14] Sterdt E Liersch S Walter U What motivates children and adolescents to be physically active? A systematic review of reviews Health Educ J 2013 10.1177/0017896912469578

[B15] Benjamin SE Neelon B Ball SC Bangdiwala SI Ammermann AS Ward DS Reliability and validity of a nutrition and physical activity environmental self-assessment for child care IJBNPA 2007 4 29 17615078 10.1186/1479-5868-4-29 PMC1934916

[B16] Ammermann AS Ward DS Benjamin SE Ball SC Sommers JK Molloy M Dodds JM An intervention to promote healthy weight: nutrition and physical activity self-assessment for child care (NAP SACC) theory and design Prev Chronic Dis 2007 4 3 [http://www.cdc.gov/pcd/issues/2007/jul/06_0115.htm] PMC1955393 17572971

[B17] Burdette HL Whitaker RC Resurrecting free play in young children: looking beyond fitness and fatness to attention, affiliation, and affect Arch Pediatr Adolesc Med 2005 159 46 50 10.1001/archpedi.159.1.46 15630057

[B18] American Academy of Pediatrics and Council on Sports Medicine and Council on School Health Active healthy living: prevention of childhood obesity through increased physical activity Pediatrics 2006 117 1834 1842 16651347 10.1542/peds.2006-0472

[B19] van Sluijs EMF McMinn AM Griffin SJ Effectiveness of interventions to promote physical activity in children and adolescents: systematic review of controlled trials Br J Sports Med 2011 42 653 657 18685076

[B20] Payr A Woll A Geuter G, Hollederer A Bewegungsförderung im Kindergarten [Physical activity promotion in kindergarten] Handbuch Bewegungsförderung und Gesundheit [Handbook physical activity promotion and health] 2012 Bern: Verlag Hans Huber 213 227

[B21] Worobey J Worobey HS Adler A Diet, activity and BMI in preschool-aged children: differences across settings Ecol Food Nutr 2005 44 455 466 10.1080/03670240500348797

